# Incidence of Ureteric strictures Following Ureteroscopic Laser Lithotripsy: Holmium:YAG Versus Thulium Fiber Laser

**DOI:** 10.5152/tud.2023.22264

**Published:** 2023-05-01

**Authors:** Sajad Ahmad Para, Mohammad Saleem Wani, Arif Hamid, Sajad Ahmad Malik, Abdul Rouf Khawaja, Saqib Mehdi

**Affiliations:** 1 Department of Urology, Sher-i-Kashmir Institute of Medical Sciences, Srinagar, India; 2 Department of Urology and Renal Transplant, Sher-i-Kashmir Institute of Medical Sciences, Srinagar, India

**Keywords:** Holmium, YAG laser, thulium fiber laser, ureteric strictures, ureteroscopy, ureteroureterostomy

## Abstract

**Objective:**

We aimed to compare the incidence of ureteric strictures between holmium:yttrium aluminum garnet and thulium fiber laser following ureteroscopic laser lithotripsy. In the present era of miniaturization of endourologic armamentarium and better optics, how safe are lasers to fire inside ureter?

**Materials and Methods:**

It is a prospective comparative study over a period of 2 years that included patients who underwent ureteroscopic laser lithotripsy for ureteric stones. Patients were randomly divided into 2 groups: group A underwent holmium:yttrium aluminum garnet laser lithotripsy and group B underwent thulium fiber laser lithotripsy.

**Results:**

A total of 478 patients were analyzed after excluding patients not willing to participate and patients lost to follow-up. Two hundred forty patients underwent holmium:yttrium aluminum garnet laser lithotripsy (group A) and 238 patients underwent thulium fiber laser lithotripsy (group B). The demographic data of 2 groups were comparable. The mean age of patients in group A and group B was 36.5 ± 12.52 years and 38.62 ± 10.71 years, respectively. The mean operative time in group A and group B was 47 ± 15 and 36 ± 13 minutes, respectively, while the mean laser time in group A and group B was 13.5 ± 45 minutes and 9.25 ± 3.2 minutes, respectively. Four (1.67%) patients in group A and 11 (4.62%) patients in group B developed ureteric strictures during follow-up, and the difference was statistically significant (*P* < .001). The mean length of stricture was 2.67 ± 1.27 cm in group A and 4.42 ± 2.2 cm in group B, and the difference was statistically significant.

**Conclusion:**

Thulium fiber laser, projected as safe laser previously, has a higher incidence of ureteric strictures compared to holmium:yttrium aluminum garnet laser when used for ureteroscopic laser lithotripsy.

**Main Points**

- Thulium fiber laser has low threshold for lithotripsy and tissue ablation compared to holmium:yttrium aluminum garnet (Ho:YAG) laser.
- Although the optical penetration depth of thulium fiber laser (TFL) is less compared to the Ho:YAG laser, the TFL energy is absorbed 16 000 times more than the Ho:YAG laser after passing 1 mm depth of tissue, reflecting into severe tissue ablation associated with TFL.
- Thulium fiber laser has a higher incidence of ureteric strictures compared to the Ho:YAG laser when used for ureteroscopic laser lithotripsy.
- Thulium fiber laser should be used cautiously inside the ureter with low energy at lower frequency setting with continuous irrigation.

## Introduction

Ureteric stones presenting as acute flank pain is a common cause of emergency department visits, and a large number of patients require definitive treatment in the form of ureteroscopic lithotripsy. Ureteroscopic lithotripsy by lasers or pneumatic lithotripters allows fragmentation of larger stones and subsequent clearance. Lasers have outclassed pneumatic lithotriptors in the management of ureteric strictures, with less retropulsion and better fragmentation o all types of stones.^1^

Holmium:yttrium aluminum garnet (Ho:YAG) laser, being an efficient lithotripter and compatible with both flexible and rigid uretroscopes, has been considered as the gold standard for ureteroscopic lithotripsy.^2^ Because of some inherent flaws in the Ho:YAG laser, thulium fiber laser (TFL) was introduced into the armamentarium of laser lithotripters to overcome these deficiencies. As a lithotripter, TFL outclasses the Ho:YAG laser in many aspects as follows: it uses fine fibers to deliver energy with core diameter as low as 50 µm; it is possible to operate TFL at modest pulse energy as low as 0.025 J; (3) it has a peak operating frequency up to 2000 Hz; (4) it has a pulsed infrared energy emission at a wavelength of 1940 nm that has a 4-fold absorption coefficient compared to the Ho:YAG laser and therefore has low threshold for lithotripsy and tissue ablation.^3^ Comparative studies have suggested faster stone ablation rates (1.5-4 times) in favor of TFL.^4^ With the use of Ho:YAG laser lithotripsy through small-size uretroscopes, the incidence of ureteric strictures was reduced compared to the use of rigid lithotripters through larger-sized uretroscopes.

Thermal effect of lasers on ureteric mucosa leads to mucosal ablation and subsequent ureteric stricture formation in many cases. Thulium fiber laser has a low depth of penetration and has been projected as safe laser for intracorporeal lithotripsy. However, studies have shown that rise in ureteric temperature was 9°C-12°C higher for TFL than the Ho:YAG laser when operated at higher frequency and slow irrigation flow, leading to increased thermal stress to the surrounding tissues.^5^ In the present study, we compared the incidence of ureteric strictures between TFL and Ho:YAG laser when used for ureteroscopic lithotripsy.

## Materials and Methods

This is a 2-year prospective comparative study, carried at our institute from January 2020 to December 2021. This study includes all the cases of ureteric stones that underwent ureteroscopic laser lithotripsy. Patients with ureteric stones were evaluated with computed tomography (CT) urography and subsequently planned for ureteroscopic laser lithotripsy. Patients were randomized to undergo lithotripsy with the Ho:YAG laser (Lumenis pulse 100H Ho laser, group A) or TFL (IPG photonics 50/500-QCW, group B). Written informed consent was taken from the patients, and ethical committee clearance was obtained to carry out this study. The study has been approved by Ethical committee of Sher-i-Kashmir Institute of Medical Sciences Srinagar Jammu and Kashmir India. Approval number is IEC/Skims protocol #231E/2020. Patients with past history of extracorporeal shockwave lithotripsy, ureteroscopy, or open surgery done on the ureter were excluded from study. Also, patients with past history of genitourinary strictures, endometriosis, retroperitoneal fibrosis, or pyonephrosis were also excluded from the study.

**Figure 1.**
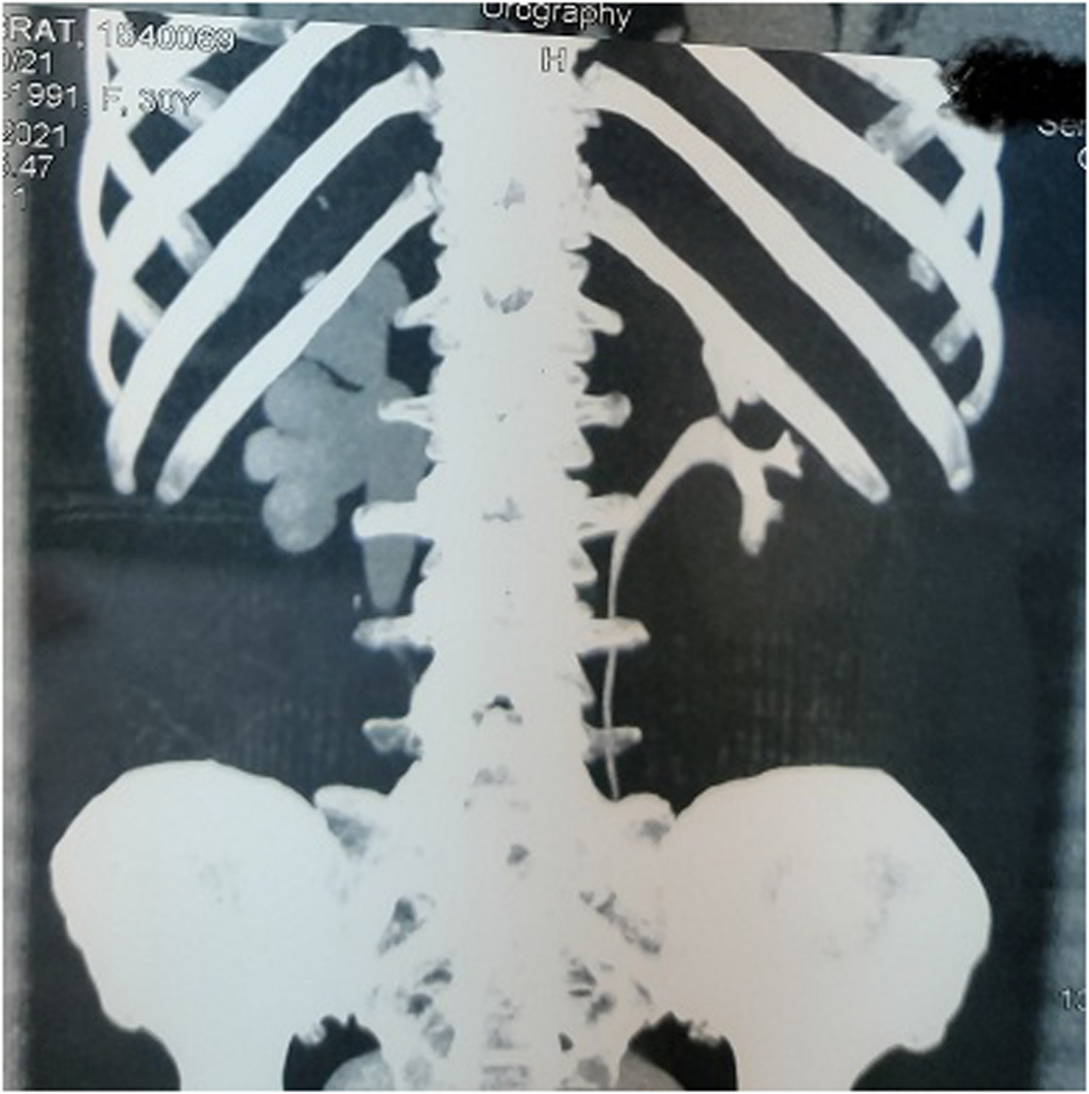
Computed tomography urography: reconstructed image showing right-sided hydroureteronephrosis due to upper ureteric stricture.

**Figure 2.**
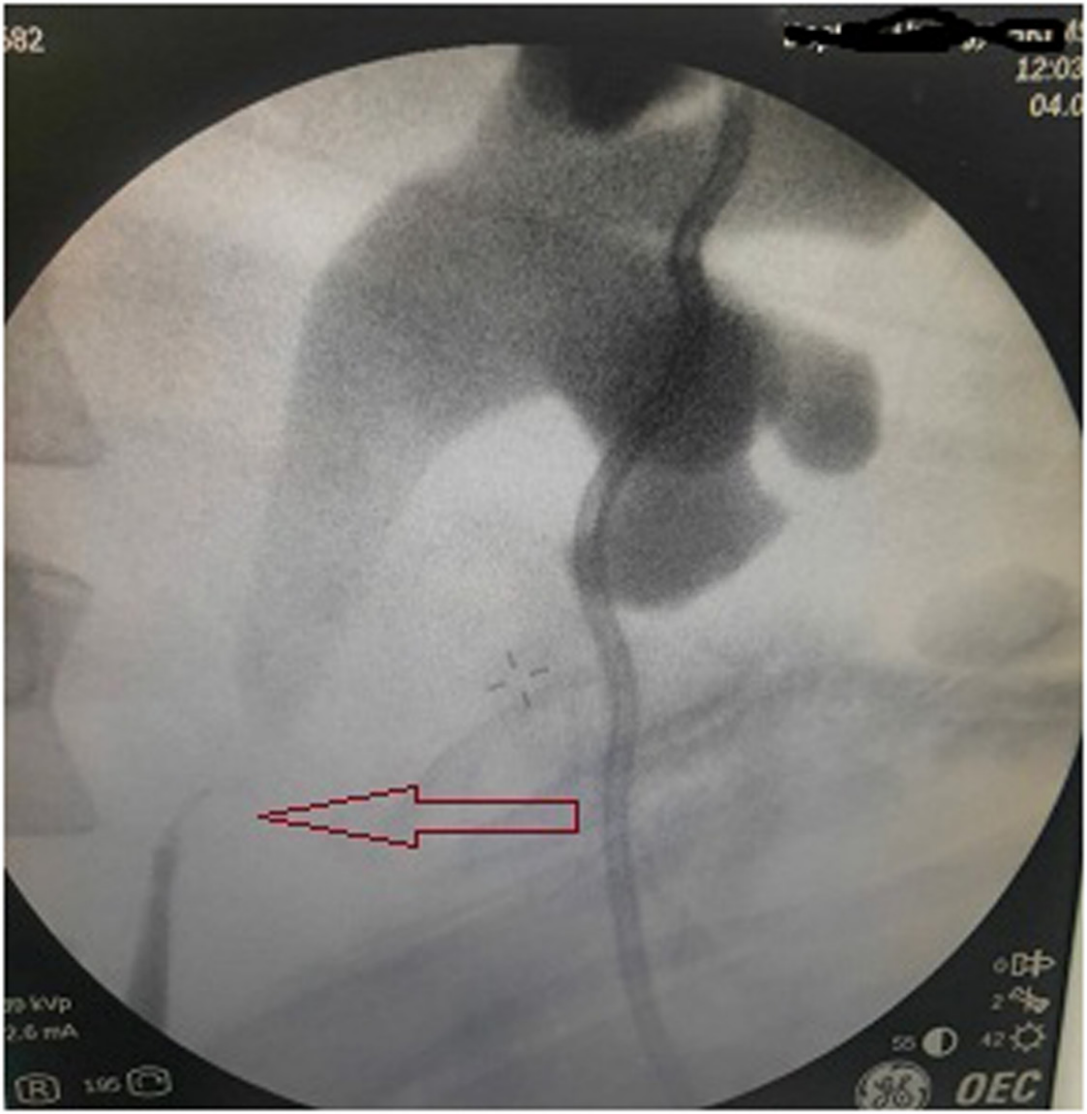
Antegrade and retrograde pyelography delineating the length of stricture (red arrow).

**Figure 3.**
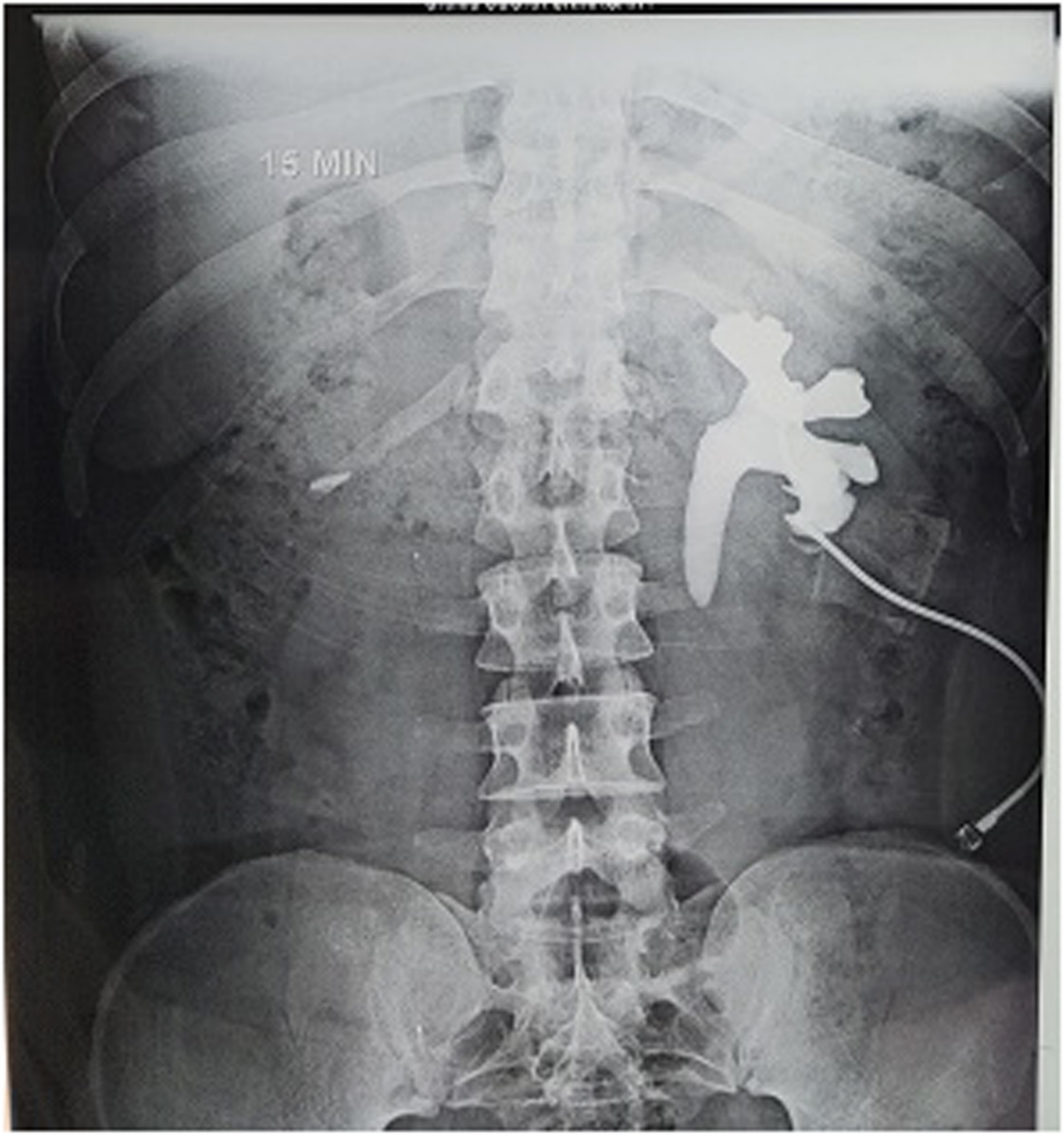
Left antegrade pyelography showing complete cutoff in the upper ureter.

**Figure 4.**
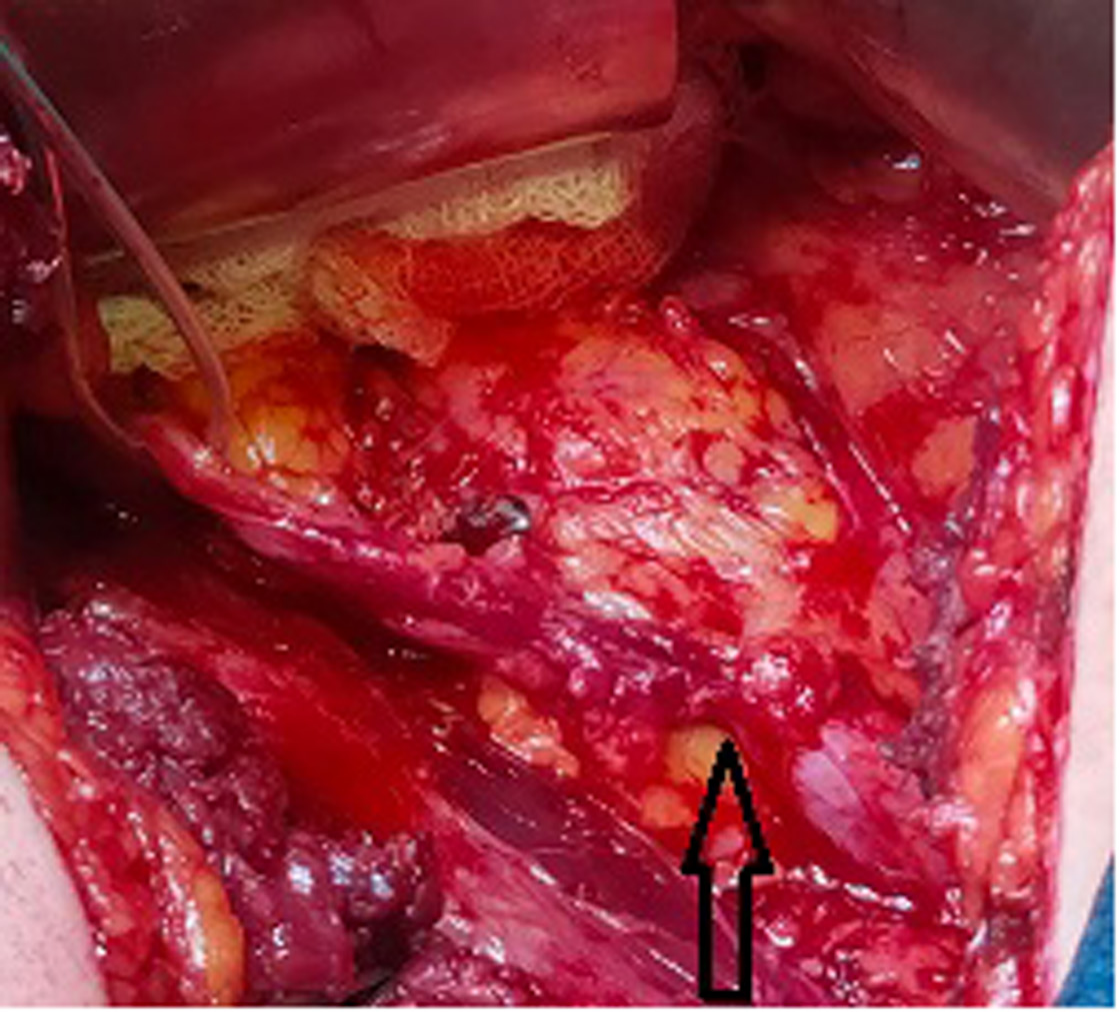
Intraoperative picture of the upper ureteric stricture (black arrow).

Ureteroscopy was done under spinal anesthesia using 6/7.5 Fr semi-rigid ureteroscope with normal saline irrigation by continuous and pulsatile irrigation bulb. The steps of the procedure were same except the type of laser used in 2 groups. In both groups 200 μm fiber was used to deliver laser energy. Lithotripsy was started with 0.4 J and 8 Hz in both the groups and gradually increased if disintegration was ineffective. After lithotripsy, larger stone fragments were removed with a forceps and a double J (DJ) stent was deployed at the end of the procedure. Stone clearance and placement of DJ stent was confirmed with fluoroscopy. A follow-up x-ray kidney ureters and bladder (KUB) was done after 2 weeks to look for any residual fragments. The DJ stent was removed 4 weeks after the procedure. An ultrasound examination of the abdomen was done 6 weeks after stent removal to look for any residual hydronephrosis. Patients with fever, flank pain, increase in hydroureteronephrosis, or obstructive uropathy underwent CT urography to identify any stricture. Patients with pyonephrosis or urosepsis underwent percutaneous nephrostomy tube placement. The length and site of stricture was estimated by CT urography (Figure 1), table retrograde pyelography (Figure 2), and antegrade pyelography (Figure 3). All those patients with ureteric strictures underwent Tc^99m^ diethylenetriamine pentaacetate (DTPA) scan to quantify renal function. Patients with ureteric strictures underwent endoureterotomy, ureteroureterostomy (Figure 4), ureteroneocystostomy, Boari flap reconstruction, or nephrectomy depending on the length of stricture, site of stricture, and salvageability of renal function. Patients treated for ureteric strictures were followed at 3 months post-surgery with Tc^99m^ DTPA scan and CT urography. Statistical analysis was done using Statistical Package for Social Sciences (SPSS) version 22.0. (IBM SPSS Corp.; Armonk, NY, USA). The qualitative data are presented as numbers and percentages, while the quantitative data are presented as mean (SD). The normal distribution of quantitative data was assessed by an independent sample *t*-test. Comparison of qualitative variables between the groups was done using the chi-square test. Statistical significance of any parameter was defined as *P*-value <.05.

## Results

**Figure 5.**
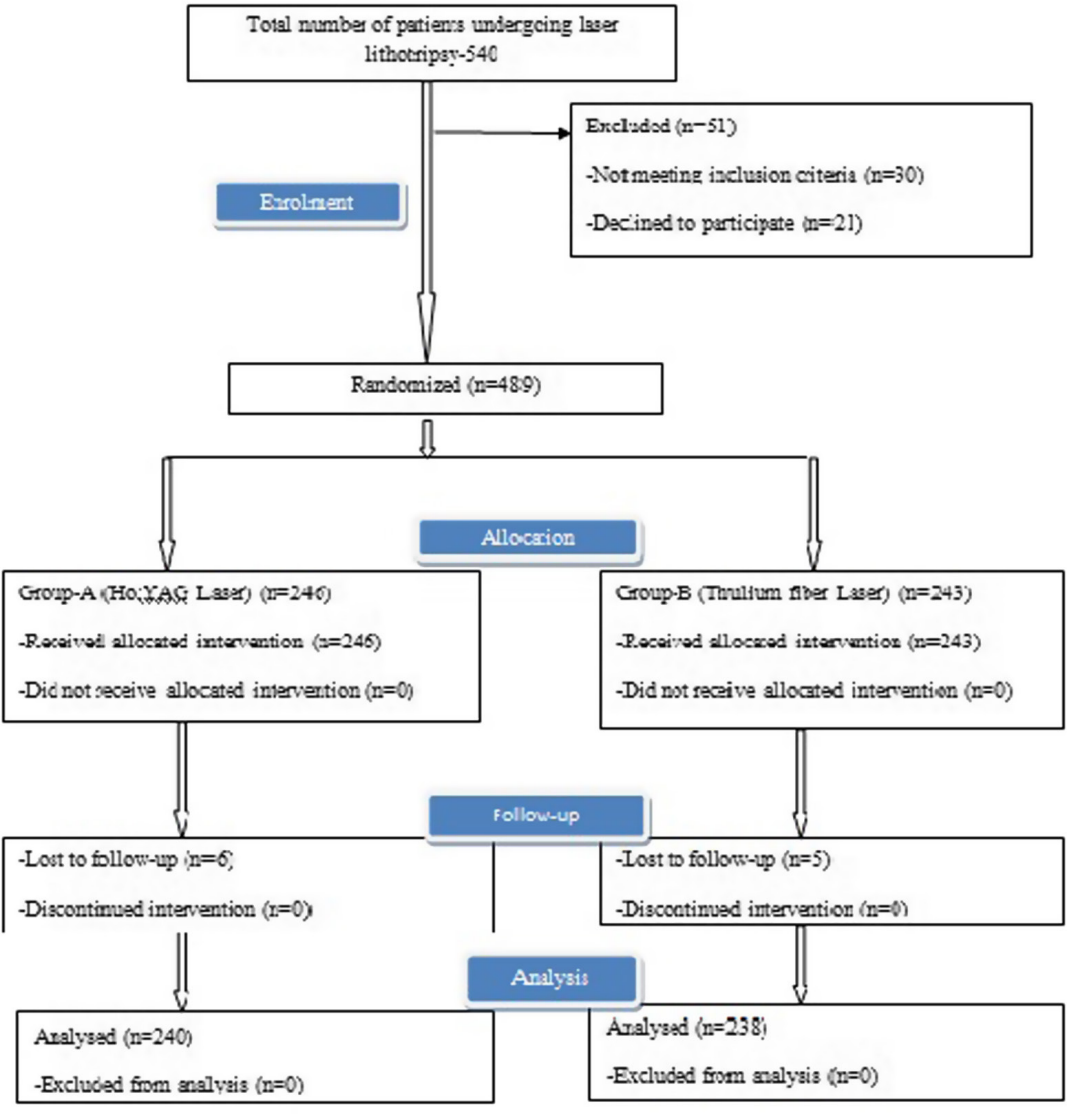
Stages of our study in a Consolidated Standards of Reporting Trials diagram.

Between January 2020 and December 2021, 540 patients with ureteric calculi were planned for ureteroscopic laser lithotripsy. Out of these 21 declined to participate and a further 30 patients were excluded as they did not qualify to participate in the study. The remaining 489 patients were randomly divided into 2 groups: group A (246 patients) who underwent Ho:YAG laser lithotripsy and group B (243 patients) who underwent TFL lithotripsy. During the study, 6 patients from group A and 5 patients from group B lost to follow-up. Figure 5 shows the stages of our study in a Consolidated Standards of Reporting Trials diagram.

The demographic profile of our study groups is shown in Table 1, and there was no statistically significant difference between the 2 groups. The percentage of females in group A and group B was 36.25% and 35.29%, respectively. The average age of patients in group A and group B was 36.5 ± 12.52 years and 38.62 ± 10.71 years, respectively, and the difference was not statistically significant (*P* = .45). The average waiting period to undergo procedure is 7.5 ± 1.5 days and 8 ± 2.5 days in group A and group B, respectively, and the difference was not statistically significant. The average stone size in group A was 8.9 ± 3.5 mm and that in group B was 9.1 ± 3.2 mm (*P* = .25). Stone density was 922 ± 241 hounsfield unit (HU) and 918 ± 235 HU in group A and group B, respectively (*P* = .82). The stone parameters of 2 groups were comparable. The percentage of stones in the upper ureter (above crossing of iliac vessels) was 40% and 39% in group A and group B, respectively (*P* = .21).

The intraoperative parameters of the 2 groups are shown in Table 2. The mean operative time in group A and group B was 47 ± 15 and 36 ± 13 minutes, respectively, and the difference was statistically significant (*P* < .05). The mean laser time in group A was 13.5 ± 4.5 minutes and that in group B it was 9.25 ± 3.2 minutes. The difference in the laser time was statistically significant (*P* < .001). Forty-eight percent of patients in group A and 37% of cases in group B required augmentation in frequency and energy to achieve the desirable fragmentation after starting with low-energy setting, and the difference was significant (*P* < .05). The average laser energy spent in group A and group B was 4.4 ± 1.2 and 3.12 ± 1.3 kJ/case, respectively, and the difference was not statistically significant (*P* < .23). Fifteen percent of patients in group A and 13.02% of patients in group B had adverse events recorded intraoperatively that included bleeding impairing vision (10% in group A and 8.82% in group B), visible mucosal ablation (8.75% in group A and 7.56% in group B), and ureteric perforation (1.25% in group A and 0.84% in group B). The difference was not statistically significant (*P* < .42).

The incidence of postoperative ureteric strictures following ureteroscopic laser lithotripsy is shown in Table 3. Four (1.67%) patients in group A and 11 (4.62%) patients in group B developed ureteric strictures during follow-up, and the difference was statistically significant (*P* < .001). Majority of patients with strictures presented with flank pain and fever (75% in group A and 82% in group B). Asymptomatic progressive hydronephrosis was reported in 1 patient of group A and 2 patients of group B. The mean time of presentation following removal of DJ stent was 41 ± 19 days in group A and 39 ± 17 days in group B, and the difference was not significant (*P* < .45). Percutaneous nephrostomy tube placement was required to drain pyonephrosis and control sepsis in 3 patients of group A and 9 patients of group B. Strictures were predominantly located in the upper ureter in both the groups (75% in group A and 78% in group B). The mean length of stricture documented on CT urography/antegrade or retrograde pyelography was 2.67 ± 1.27 cm in group A and 4.42 ± 2.2 cm in group B, and the difference was statistically significant (*P* < .001).

Laser endoureterotomy was done to treat small passable strictures. One patient in each group had strictures amenable to laser endoureterotomy. Two patients in group A and 5 patients in group B required ureteroureterostomy to restore continuity of ureter. Short-segment lower ureteric strictures, 1 in group A and 2 in group B, required ureteroneocystostomy. One patient in group B with long-segment lower ureteric stricture required Boari flap reconstruction. Two patients in group B required nephrectomy for non-functional kidney; however, no nephrectomy was reported in group A.

## Discussion

Ureteroscopic lithotripsy is considered as the gold standard treatment for ureteric stones. Conventional lithotripsy by pneumatic lithotripters had the problem of poor fragmentation of harder stones and retropulsion. Introduction of laser lithotripters has allowed the use of small-size uretroscopes, better vision because of good flow of irrigation fluid through ureteroscope, efficient fragmentation, and less retropulsion, thus reducing the operative time and the incidence of ureteric strictures.^6^ Holmium:YAG laser became the gold standard in laser lithotripsy with its ability to fragment all kinds of stones and a better safety profile. Safety was attributed to its limited tissue penetration and high absorption coefficient in water, thus reducing the collateral damage.^7^

There are some flaws associated with the Ho:YAG laser generator. The generator requires an adequate cooling system that contributes to its large size. High-power Ho:YAG generator employs numerous crystal cavities to integrate the overall power output. These architectural anomalies result in an output beam that is multimodal and nonuniform with hotspots. This type of laser beam is difficult to precisely focus onto a small target and therefore demands the use of thicker optical fiber with a core diameter of 200 µm or larger.^8^ The architecture of the Ho:YAG generator makes it susceptible to external shocks, leading to the misalignment of reflecting mirrors within the crystal cavity causing enduring damage to the generator and the optical fiber. These limitations paved the way for TFL into intracorporeal lithotripsy. The TFL uses a thin (10-20 µm core diameter) and long silica fiber doped with thulium ions. Multiple diodes are used to excite thulium ions. The final output beam has a wavelength of 1940 nm that can be operated in continuous or pulsed mode. The diode laser used for laser pumping has an emission spectrum that precisely matches the thulium ion absorption line. There is less heat dissipation and a potential to operate at high-power setting (>50 W) and higher frequencies (up to 2000 Hz). It uses small-sized fans for forced air cooling, reducing the size of the machine.^9^

Thulium fiber laser is an efficient lithotripter with 2 times faster fragmentation and 4 times superior dusting compared to the Ho:YAG laser. Schembri et al^10^ reported that TFL attains high ablation rates and surpasses Ho:YAG laser over a range of different settings and ablation modes. The ability of TFL to operate at low pulse energy reduces retropulsion and need of ancillary procedures for residual stones. In our study, the mean operative time and mean laser time were less in the TFL group compared to the Ho:YAG laser group, and the difference was statistically significant. Besides efficient lithotripsy, better vision and less retropulsion by TFL may be the reason for less operative time compared to the Ho:YAG laser. The mean laser energy spent in lithotripsy did not differ in the 2 groups. Stone factors like density, composition, and size of the stone determine the mean laser energy spent during the procedure, and these factors were comparable in the 2 groups.

There is a significant variability about the ureteric stricture rate following ureteroscopic lithotripsy reported in the literature, with incidence ranging from 0.30% to 23.81%. Adiyat et al^11^ and Li et al reported a stricture rate of 1.4% and 2.95%, respectively, following Ho:YAG laser lithotripsy, and the results are comparable with our study. The incidence of ureteric strictures in our study was reported to be higher in the TFL group as compared to the Ho:YAG laser group, and the difference was statistically significant. The TFL used for the procedure in our study was IPG photonics 50/500-QCW TFL. The TFL beam has a wavelength of 1940 nm, which is equivalent to the near-infrared absorption peak of water at 22°C. The absorption coefficient of the TFL is 14 mm^−1^, which corresponds to the optical penetration depth of 0.077 mm in water and is much less than that of the Ho:YAG laser having an optical penetration depth of 0.4 mm.^12^ These features should add to the safety profile of the TFL, but what is the reason for clustering of stricture cases in our study that is predominantly related to TFL use? The answer needs to be dug out.

The intensity of the laser beam diminishes while travelling through a medium because of absorption, and the rate of decay in intensity is determined by the absorption coefficient of the material *α*. According to Beer–Lambert law, the intensity of laser beam diminishes exponentially with the depth as it travels through the medium.^13^

*I_z_* = I_0_e^−^*^αz^*

where *I_z_* is the intensity after penetrating the depth *z*, *z* is the depth penetrated, *e* (*e* = 2.7182) is the Euler’s number, and *α* is the absorption coefficient of the material *α*.

According to this mathematical equation, the energy of a laser beam will decay by a factor of *e* (2.71828) while traversing each successive optical penetration depth of that medium. The energy of the Ho:YAG laser beam will be reduced to 37% of its energy at origin after travelling through a medium for a distance corresponding to its optical penetration depth. For TFL, energy will reduce to 1.7% of its energy at source after travelling the distance equivalent to the optical penetration depth of that medium. Practically after traversing 1 mm depth of water, the Ho:YAG laser pulse will possess about 4% of its energy at source, while the TFL beam will have a mere intensity of 0.00024% left with it after travelling the same distance. No doubt, the optical penetration depth of TFL is less compared to the Ho:YAG laser. Because of the logarithmical pattern of laser energy absorption in the tissue, the TFL energy is absorbed 16 000 times more than the Ho:YAG laser after travelling 1 mm depth. This high absorption coefficient reflects in low threshold for tissue ablation and more tissue damage in favor of TFL. The TFL has a natural “moses” potential attributed to its uniform pulse energy.^14^ The depth traversed is again augmented by the “Moses” effect. The TFL pulse has a low threshold for tissue ablation and vapor channel initiation because of its high water absorption coefficient that translates into worse tissue damage in the ureter during laser lithotripsy.^15^

Another factor suggested for more stricture rate is the high temperature generated during TFL laser lithotripsy. Ureteric temperatures have exceeded physiological limits after TFL use at high-energy setting and low irrigation rate.^16^ Lack of ureteric access sheath and poor return of irrigation fluid in rigid ureteroscopy are the obvious reasons for raised intrauretric temperature. The threshold temperature for cellular damage being 43°C is crossed within the first 1 second of laser use in the absence of adequate irrigation. Surgical factors increasing the chance of thermal ureteric injury are low irrigation flow, higher laser energy setting, and instrument use without access sheath.^17^ Liang et al^18^ reported a marked rise in the ureteric temperature at higher frequency compared to lower frequency setting at equal power. So these might be contributing factors to the high stricture rate in the TFL group. Tokas et al^19^ studied the effect of rise in the intrauretric temperature during laser lithotripsy and its effect on the course of healing. At higher energy settings, TFL causes a marked rise in intrauretric temperature and worse tissue damage reflecting into dense scars on subsequent healing. They proposed brief on/off laser activation intervals, cold continuous irrigation, and use of access sheaths for better irrigation to maintain physiological intrauretric temperature.

The average length of stricture in our study was 2.67 ± 1.27 cm in the Ho:YAG group and 4.42 ± 2.2 cm in the TFL group. Larger stricture length in the TFL group can be attributed to low threshold for tissue ablation and more pronounced rise in the intrauretric temperature. Small-length upper ureteric strictures are well managed by ureteroureterostomy with excellent results.^20^ Two patients in group A and 5 patients in group B needed ureteroureterostomy to restore the continuity of ureter. Laser endoureterotomy has a success rate of 62% for small-length ureteric strictures.^21^ One patient in each group underwent laser endoureterotomy with successful outcome. Small-segment lower ureteric strictures are amenable to ureteroneocystostomy, while long-segment lower ureteric strictures require Boari flap reconstruction.^22^ One patient in the Ho:YAG group and 2 in the TFL group required ureteroneocystostomy, while 1 patient in the TFL group required Boari flap reconstruction for long-segment lower ureteric stricture. Non-salvageable renal function with pyonephrosis was managed by nephrectomy in the TFL group.

## Conclusion

There is a higher incidence of ureteric strictures following the use of TFL for ureteroscopic lithotripsy. Although projected as a safe laser, TFL should be used cautiously inside the ureter especially at higher power/frequency settings. Low energy at low frequency setting with continuous irrigation can reduce the incidence of ureteric strictures in TFL lithotripsy.

## Figures and Tables

**Table 1.** Demographic Profile of Patients

Demographic Profile	Group A (Ho:YAG), n = 240	Group B (TFL), n = 238	*P*
Age in years (mean ± SD)	36.5 ± 12.52	38.62 ± 10.71	.45
Sex (male/female)	153/87	154/84	.28
Stone size in mm (mean ± SD)	8.9 ± 3.5	9.1 ± 3.2	.25
Stone density in HU (mean ± SD)	922 ± 241	918 ± 235	.82
Location of stone in percent (upper ureter/lower ureter)	40/60	39/61	.21

Ho, holmium; TFL, thulium fiber laser; YAG, yttrium aluminum garnet.

**Table 2.** Intraoperative Parameters of the 2 Study Groups

Intraoperative Parameters	Group A (Ho:YAG), n = 240	Group B (TFL), n = 238	*P*
Operative time in minutes (mean ± SD)	47 ± 15	36 ± 13	.05
Laser time in minutes (mean ± SD)	13.5 ± 4.5	9.25 ± 3.2	.001
Laser energy spent in kJ/case (mean ± SD)	4.4 ± 1.2	3.12 ± 1.3	.23
Adverse events recorded (%) –Bleeding impairing vision –Visible mucosal ablation –Ureteric perforation	15% 10% 8.75% 1.25%	13.02% 8.82% 7.56% 0.84%	.42

Ho, holmium; TFL, thulium fiber laser; YAG, yttrium aluminum garnet.

**Table 3.** Comparison of Ureteric Strictures in 2 Study Groups

Ureteric Strictures Following Procedure	Group A (Ho:YAG), n = 240	Group B (TFL), n = 238	*P*
Incidence of ureteric strictures, n (%)	4 (1.67%)	11 (4.62%)	.001
Presenting symptom, n (%) –Fever and pain –Asymptomatic progressive hydronephrosis	3 (75%) 1 (25%)	9 (82%) 2 (18%)	.22
Time of presentation following the removal of DJ stent, in days (mean ± SD)	41 ± 19	39 ± 17	.45.
Percutaneous nephrostomy tube placed, n (%)	3 (75%)	9 (81.81%)	.42
Location of stricture, n (%) –Upper ureter –Lower ureter	3 (75%) 1 (25)	8 (72.72%) 3 (27.27%)	.35
Mean length of stricture in cm (mean ± SD)	2.67 ± 1.27	4.42 ± 2.2	.001
Procedures done for ureteric strictures –Laser endoureterotomy –Ureteroureterostomy –Ureteroneocystostomy –Boari flap reconstruction –Nephrectomy	1 2 1 0 0	1 5 2 1 2	

DJ, double J; Ho, holmium; TFL, thulium fiber laser; YAG, yttrium aluminum garnet.
